# Methane emissions and rumen microbiome response during compensatory growth on either a forage or grain-based finishing diet in beef cattle

**DOI:** 10.1093/tas/txae143

**Published:** 2024-09-27

**Authors:** Juan M Clariget, Georgget Banchero, Verónica Ciganda, Daniel Santander, Kate Keogh, Paul E Smith, Alan K Kelly, David A Kenny

**Affiliations:** Instituto Nacional de Investigación Agropecuaria, Colonia, Uruguay; Teagasc Animal and Grassland Research and Innovation Centre, Dunsany, Ireland; School of Agriculture and Food Science, University College Dublin, Belfield, Ireland; Instituto Nacional de Investigación Agropecuaria, Colonia, Uruguay; Instituto Nacional de Investigación Agropecuaria, Colonia, Uruguay; Instituto Nacional de Investigación Agropecuaria, Colonia, Uruguay; Teagasc Animal and Grassland Research and Innovation Centre, Dunsany, Ireland; Teagasc Animal and Grassland Research and Innovation Centre, Dunsany, Ireland; School of Agriculture and Food Science, University College Dublin, Belfield, Ireland; Teagasc Animal and Grassland Research and Innovation Centre, Dunsany, Ireland

**Keywords:** compensatory gain, finishing, greenhouse gases, microbiota, restriction

## Abstract

The aim of this experiment was to evaluate the effect of the level of prior nutritional restriction during backgrounding in Angus steers on methane (CH_4_) emissions, diet digestibility, rumen fermentation, and ruminal microbiome under either a forage or grain-based finishing diet. Eighty steers (body weight [BW]: 444 ± 39 kg, age: 18 ± 1 mo) were blocked and randomly assigned within the block to either an **optimal** (0.6 to 0.7 kg/d) or **suboptimal** (0.3 to 0.4 kg/d) growth rate to exploit compensatory growth (CG), during 97 d of backgrounding. Following, for 84 d, half of the steers in each group were finished on a **forage** diet while the other half were finished on a **grain**-based diet. During the backgrounding period, CH_4_ emissions tended (*P* ≤ 0.07) to be higher; however, CH_4_ intensity expressed by BW gain was 50% lower (*P* < 0.01) for optimal compared to suboptimal growth steers. BW gain, dry matter intake, diet digestibility, and ammonia nitrogen in the rumen were greater (*P* < 0.01) for optimal compared to suboptimal steers. During the finishing period, CH_4_ emissions in either forage or grain finishing diets were similar (*P* > 0.05) for both backgrounding treatments. However, due to greater BW gain in suboptimal steers (1.20 vs. 0.97 kg/d), their CH_4_ intensity-related coefficient decreased (*P* < 0.05) during the finishing period. Diet digestibility or any fermentation parameter was unaffected (*P* > 0.05) by previous backgrounding during the finishing period. In fact, rumen microbial abundance measured during finishing was not modified (*P* > 0.05) by previous backgrounding. Steers finished under grain conditions, had lower (*P* < 0.01) daily CH_4_ emissions and CH_4_ intensity. Additionally, grain-fed steers increased (*P* < 0.05) BW gain, diet digestibility, propionic, lactic, and valeric acids, *Succinivibrionaceae* family and *Succiniclasticum*, *Erysipelotrichaceae UCG-002*, *Sharpea*, and *Megasphaera* bacteria genera, compared to forage-fed steers. In conclusion, ruminal microbiome and fermentation, diet digestibility, and CH_4_ emissions were unaffected during finishing between prior levels of backgrounding growth. However, given the higher BW gain in suboptimal steers in both finishing diets, CH_4_ intensity was reduced in comparison to the optimal backgrounded steers. Nevertheless, lifetime emissions of the steers need to be assessed with the different dietary regimens, since suboptimal steers reduced CH_4_ emissions during the backgrounding period but, additional days of finishing were required to achieve the same BW as their contemporaries.

## Teaser

Either under forage or grain-based finishing regimens, steers initially subjected to a suboptimal growth during backgrounding had lower methane intensities (g CH_4_ /kg BW gain and g CH_4_ /kg HCW gain). Nevertheless, this was due to their relatively higher performance during the finishing phase, rather than lower methane emissions or methane yield per se. Lifetime emissions of the steers need to be assessed with the different dietary regimens, since suboptimal steers reduced CH_4_ emissions during the backgrounding period but, additional days of finishing were required to achieve the same BW as their contemporaries.

## Graphical Abstract

**Graphical Abstract:**
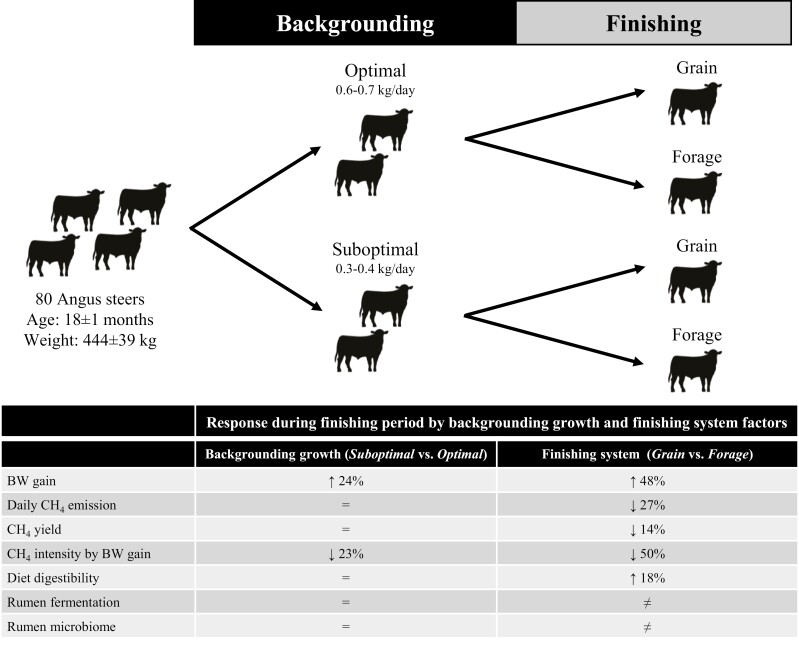

## Introduction

Ruminant livestock, with their unique ability to convert lignocellulolytic plant matter into a high-quality source of dairy and meat protein, are an integral part of the global food system (Waters et al., 2020). However, the potent greenhouse gas (GHG) methane (CH_4_) is produced as a byproduct of the ruminal fermentation of feed (Beauchemin et al., 2020). Due to a combination of global demand for ruminant animal-derived protein, and the environmental potency of enteric CH_4_, ruminant livestock are directly accountable for an estimated 5% of anthropogenic GHG emissions (Crippa et al., 2021).

The exploitation of compensatory growth (CG), whereby after a period of restricted feeding, an animal has the potential to undergo enhanced growth upon realimentation (Keogh et al., 2015) can improve the lifetime feed efficiency of an animal (Keady et al., 2021). However, the mechanism associated with the improvement of feed efficiency during realimentation, like digestion and kinetics fermentation is unclear with diverse results in the literature (Choat et al., 2003; McCabe et al., 2015; Silva et al., 2020). Additionally, there is currently a lack of published data on enteric CH_4_ output, during a period of realimentation either at forage or grain finishing induced CG in beef cattle. So, a comparison of diet digestibility, rumen fermentation, and microbial profiles were conducted in an attempt to investigate the underlying biological processes associated with CH_4_ emissions in animals that underwent feed restriction and subsequent CG. Also, subjecting animals to a period of feed restriction during backgrounding in the beef farm system has been shown to increase slaughter age (Costa et al., 2015; Peripolli et al., 2017; Mezgebo et al., 2018); so, it is imperative that the effects of dietary realimentation on enteric CH_4_ emissions are defined, to holistically assess the impact of feed restriction on the overall lifetime emissions of beef cattle. Therefore, the aim of this experiment was to evaluate the effect of the level of prior nutritional restriction during backgrounding in Angus steers on CH_4_ emissions, diet digestibility, rumen fermentation, and ruminal microbiome under either a forage or grain-based finishing diet.

## Materials and Methods

The experiment was conducted at the livestock unit of the National Institute of Agricultural Research (INIA), at La Estanzuela Research Station, Uruguay (57°42ʹ54.00″ W latitude; 34°20ʹ46.56″ S longitude). All experimental procedures conducted on animals in this study were approved by the Committee for the Ethical Use of Animals of the National Institute of Agricultural Research, Uruguay (INIA, Protocol number 2020-5).

### Experimental Design and Treatments

One hundred and twelve Angus calves were purchased at 8 ± 1 mo of age from a single commercial herd and managed together on the same diet until the start of the experiment. Prior to the start of the experimental treatments, the steers were acclimatized to their pens and electronic feeders (INTERGADO, Minas Gerais, Brazil) for 47 d with a diet that consisted of ad libitum grass haylage. At an average age of 18 ± 1 mo and body weight (BW) of 444 ± 39 kg, steers were blocked by BW, hip height, and daily gain during the grazing period, and randomly assigned within the block to 1 of the 4 treatments. The experiment was a randomized complete block design with a 2 × 2 factorial structure. One factor was the growth rate, either optimal or suboptimal during the backgrounding period for the subsequent expression of CG, meanwhile, the other factor was the finishing diet, forage, or grain-based. Optimal growth rate during backgrounding was defined as a daily gain between 0.6 and 0.7 kg/d; meanwhile, suboptimal backgrounding growth rate was defined as a daily gain between 0.3 and 0.4 kg/d. Finishing diets were forage-based or grain-based (75% concentrate). The backgrounding period lasted 97 d, while the finishing period lasted 84 d. Of the total 112 steers used for a larger study, 80 steers were selected for this specific study (20 steers per treatment), which focused on CH_4_ emissions, diet digestibility, rumen fermentation, and ruminal microbiome.

### Cattle Facilities, Feed Management, and Feed Analyses

The steers used for the current study were distributed evenly according to treatment across three different pens. Each pen had four INTERGADO automatic feeders (Oliveira et al., 2018) with access to a limiting door. One feeder per pen was used per each treatment. The limiting door can be opened by the steer when the EID tag of each steer corresponds to that feeder; otherwise, the door remains closed, and the steers cannot access the feed in the feeder. Feedstuffs were offered on an ad libitum basis (daily refusal >5%). The diets during the backgrounding period consisted of 100% alfalfa + orchard-grass haylage (optimal) vs. 70% alfalfa + orchard-grass haylage plus 30% barley straw (suboptimal). During the finishing period, diets consisted of 100% alfalfa + orchard-grass haylage (forage) vs. 75% concentrate (70% high-moisture corn grain + 5% protein-vitamin-mineral supplement) plus 25% alfalfa + orchard-grass haylage (grain).

Dry matter (DM; Harris, 1970), crude protein (CP; AOAC, 1990), neutral detergent fiber (NDF; Van Soest et al., 1991), gross energy (GE; with an adiabatic bomb calorimeter -Autobomb Gallenkamp-), total digestible nutrients (TDN; Weiss et al., 1992), and metabolizable energy, net energy for maintenance and net energy for gain were estimated based on the formulas recommended by NASEM (2016). Values for optimal vs. suboptimal diets during the backgrounding period were: 57.9% vs. 63.1% DM, 14.8% vs. 12.1% CP, 47.8% vs. 54.9% NDF, 4.27 vs. 4.19 Mcal/kg DM, 56.0% vs. 52.1% TDN, 2.07 vs. 1.92 Mcal/kg DM, 1.23 vs. 1.11 Mcal/kg DM, 0.69 vs. 0.58 Mcal/kg DM, respectively. Additionally, values for grain vs. forage-based diets during the finishing period were: 65.7% vs. 60.5% DM, 13.4% vs. 17.4% CP, 18.6% vs. 41.1% NDF, 4.32 vs. 4.15 Mcal/kg DM, 76.4% vs. 57.9% TDN, 2.83 vs. 2.14 Mcal/kg DM, 1.88 vs. 1.29 Mcal/kg DM, 1.24 vs. 0.74 Mcal/kg DM, respectively.

### Methane Emissions

For the measurement of CH_4_, the sulfur hexafluoride (SF_6_) tracer gas technique (Johnson et al., 1994; Gere and Gratton, 2010) was used in nine steers from each treatment combination (a total of 36 animals). The CH_4_ gas was collected on two occasions: at the end of the backgrounding period (days 79 to 85), and at the end of the finishing period (days 161 to 167). For practical reasons, CH_4_ was determined with a 1-d phase shift between pen to pen, to distribute the work more evenly and not overload different measurements in a single day. Seven days before the beginning of the measurements, 36 steers were given an oral permeation tube filled with SF_6_ using a stainless-steel oral dosing applicator. The tube was used as a marker for gas emissions. The tubes were incubated in the water bath at 39 °C for 28 d before being used. Initial permeation rates of SF_6_ from the tubes provided were an average of 6.57 ± 1.13 mg/d. After slaughter, the tubes were removed from the rumen, and incubated at 39 °C for 28 d, and the permeation rate was calculated again with an average of 6.05 ± 1.29 mg/d.

The collection of exhaled and eructed gas was performed using two 0.5-L stainless evacuated steel containers per animal. These containers were used as gas collectors coupled to a muzzle and placed on each side of the back using a specialized harness. Following the procedure performed by other authors (Pinares-Patiño et al., 2012; Dini et al., 2018, 2019), the containers remained on each animal for 5 d. At the end of each sampling period, the containers were removed from the animals and post-sampling pressure was measured. Containers with pressure values of 400 to 600 mb were considered valid according to Gere and Gratton (2010) and Gere (2012) ensuring good quality samples. Containers outside of this range of pressure values were removed from the experiment (15% removed). Two subsamples were extracted from each container and stored in 6-mL vacutainers for the determination of CH_4_ and SF_6_ concentrations. Methane and SF_6_ ambient concentration baselines were obtained through the use of three collection containers placed around each pen during each recording phase.

A gas chromatograph (Agilent 7890A, Santa Clara, CA) was used for the analysis, with a flame ionization detector and an electron capture detector for determining CH_4_ and SF_6_ concentrations, respectively. After obtaining a chromatographic analysis of samples, CH_4_ and SF_6_ were corrected by multiplying the concentration by the correction factor. The correction factor was calculated by dividing the final pressure by the initial pressure of each container. Finally, once the concentrations of enteric CH_4_ and SF_6_ (CH_4_ ent and SF_6_ ent) of the subsamples collected from the animals and the concentrations of the ambient container (CH_4_ amb and SF_6_ amb) were corrected, the daily emission of enteric CH_4_ was calculated using the following equation, which includes a correction for the molecular weights of each gas:


$$\begin{array}{lll} CH4\;(g/d)= & \;SF6\;PR\;(mg/d)*\left[\frac{CH4\;ent\;-CH4\;amb\;(ppm)}{SF6\;ent\;-\;SF6\;amb\;(ppt)}\right]\; & *\left[\frac{16.04\;g/mol\;CH4}{146.06\;g/mol\;SF6}\right]*1000 \end{array}$$

Samples were considered accurate if the coefficient of variation within the tube and between tubes from the same animal was less than 20%. If the variation was higher than 20%, the data were eliminated (3% eliminated).

Methane yield was expressed by DM intake (DMI), meanwhile, CH_4_ conversion rate (Ym) was calculated by the fraction of GE intake (GEI) that is converted to CH_4_. Methane intensities were expressed per unit of BW, BW gain, and HCW gain. For CH_4_ yield and Ym factor, the specific intake on the same 5 d that CH_4_ was recorded, DMI was measured. For the expression on BW, the nearest previous weight to when the CH_4_ measurements were collected was used, and for the expression relative to BW gain and HCW gain, the average emission recorded for each period was considered.

### Performance and Slaughter Measurements

Daily feed intake (wet basis) for the 36 steers that had CH_4_ measured was reported individually on an INTERGADO system using the individual ear tag and the electronic scales of each feeder. Feeding events were recorded with each 25 g fluctuation in weight at the feed bunk. DMI (kg DM/d) was calculated from the wet basis feed intake multiplied by its DM content. Steer BW was recorded every 14 d. Weighing was performed in the morning at the same hour, before the first meal (0630 hours) was provided. BW gain (kg/d) was calculated as a linear regression of the BW slope for each steer.

The 36 steers used for CH_4_ emissions measurements were slaughtered in a commercial slaughterhouse after the finishing period. They were loaded and transported (15 km, 0.5 h) to the slaughter facility on the previous day (1800 hours). The carcass was weighed after dressing (hot carcass weight, HCW; kg). The average daily gain of HCW (kg/d) was calculated as the difference between the final carcass weight less the initial carcass weight. Initial carcass weights were estimated by performing a linear regression of the slope of each treatment group using the BW and the carcass weights of the other 32 representative steers serial slaughtered at the beginning of the experiment and at the end of the backgrounding period (16 steers slaughtered as a pretrial group and 16 steers slaughtered [8 optimal and 8 suboptimal] at the end of backgrounding period).

### Diet Digestibility

Diet DM digestibility was estimated on two occasions on the 80 steers, at the end of the backgrounding period (days 82 to 86) and at the end of the finishing period (days 164 to 168). Fecal grab samples were obtained via rectal palpation once daily before morning feeding over five consecutive days. During fecal grab collection, the feed offered on each feeder was also daily taken. Fecal grab and feed samples were dried in a forced-air oven at 60 °C for 96 and 48 h, respectively, according to Harris (1970). A pool per animal of the 5 d of fecal grab sampling and another pool per feeder of the 5 d of feed sampling was made. The concentration of acid insoluble ash (AIA) was analyzed on both sample types. The AIA marker technique, as described by Van Keulen and Young (1977) and reviewed by Sales and Janssen (2003) was used, for the estimation of DM digestibility (%).

### Rumen Fluid Collection

On the same day that the SF_6_ apparatus was removed (days between 83 and 85 and days between 165 and 167), samples of rumen fluid were obtained from the 80 steers, using a transoesophageal rumen sampling device (FLORA rumen scoop; München, Germany) as previously described by Geishauser et al. (2012) and Smith et al. (2021). Samples of ~20 mL of rumen fluid were obtained from the 80 steers, at the end of the backgrounding period (days between 83 and 85) and at the end of the finishing period (days between 165 and 167). Feed was restricted from animals for a minimum of 1 h prior to sampling. After collection, ruminal fluid pH was measured immediately using a digital pH meter (Oakton, Sigma-Aldrich, Singapore). Subsequently, 2 × 1 mL subsamples were obtained to determine the concentrations of volatile fatty acids (VFA) and for rumen microbial analysis. Another sub-sample of rumen fluid (8 mL) was preserved in sulfuric acid (50%, v/v) in a 50:1 ratio, to determine the concentration of ammonia nitrogen (N-NH_3_). After collection and preparation, all rumen samples were snap-frozen in liquid nitrogen. On the same day of sampling, samples were transported 3 km to the laboratory facility and subsequently stored at −80 °C until further analysis was conducted.

### Rumen Metabolite Analysis

For determination of VFA concentration in the 80 steers, samples were thawed at room temperature and subsequently centrifuged at 13,500 rpm at 4 °C for 15 min. The concentration of VFA were measured by HPLC (Dionex Ultimate 3000, Waltham, MA, USA) with an Acclaim Rezex Organic Acid H^+^ (8%) and a 7.8 × 300 mm column (Phenomenex, Torrance, CA, USA) at 210 nm, as described by Adams et al. (1984). Samples were injected using an automated autosampler set with an injection volume of 20 μL. The concentrations of lactic, acetic, propionic, butyric, and valeric acids were determined using calibration curves for each acid with pure authentic standards (Sigma-Aldrich). The total VFA (mM) of ruminal fluid was determined as the sum of acetic, propionic, butyric, and valeric acids. The proportion of each acid in the total concentration was calculated, and the relationship between acetic and propionic acid as well as the ratio between (acetic + butyric)/propionic was also included. The determination of N-NH_3_ in ruminal liquid was carried out by direct distillation using the Kjeldahl method (Galyean, 2010) and expressed in mg N/dL of rumen fluid.

### Rumen Microbial DNA Extraction and Sequencing

Rumen microbial analysis was performed on a total of 60 samples (*n* = 15 samples per treatment) obtained during the finishing period (days between 165 and 167) of the four treatments: optimal-grain, optimal-forage, suboptimal-grain, and suboptimal-forage. Rumen microbial DNA extraction, 16S rRNA library preparation, and sequencing were outsourced to a commercial sequencing company (Macrogen; Seoul, South Korea). Briefly, microbial DNA was extracted from 500 μL of rumen fluid using the Qiagen DNeasy PowerSoil Pro Kit (Qiagen, Manchester, United Kingdom) and stored at −80 °C. Microbial DNA extractions were also performed on the ZymoBIOMICS Microbial Community Standard (MC; Zymo Research Corp., Irvine, CA, USA), for each extraction kit (*n* = 3), as an internal positive control.

Using 12.5 ng of extracted rumen microbial DNA, amplicon libraries (*n* = 63) were generated by performing two rounds of PCR amplification as outlined in the Illumina Miseq 16S Sample Preparation Guide. The first round of PCR amplification, targeting the V4 hypervariable region of the 16S rRNA gene, was performed using the 515F/806R primers (Caporaso et al., 2011) designed with Nextera overhang adapters and Herculase II Fusion DNA Polymerase (Agilent, Santa Clara, CA, USA). Cycle conditions were as follows: 95 °C for 3 min, 25 cycles at 95 °C for 30 s, 55 °C for 30 s, 72 °C for 30 s, and then 72 °C for 5 min. PCR amplicon purification was performed using the standard AMpure paramagnetic bead protocol (Beckman Coulter, Indianapolis, IN, USA). Following purification, amplicons were subjected to a second round of PCR to permit the attachment of dual indices and Illumina sequencing adapters using the Nextera XT indexing kit (Illumina, San Diego, CA, USA). Cycle conditions for the second round of PCR were 95 °C for 3 min, 10 cycles at 95 °C for 30 s, 55 °C for 30 s, 72 °C for 30 s, and then 72 °C for 5 min followed by an additional PCR purification with the AMpure paramagnetic bead protocol (Beckman Coulter, Indianapolis, IN, USA). Amplicons were pooled together in equal concentration, and subject to sequencing on the Illumina MiSeq using the 500-cycle version 2 MiSeq reagent kit (Illumina) on a single flow cell.

### Sequencing Analysis

Amplicon sequence data were processed in R (version 4.2.0) using DADA2 (version 1.26.0) and submitted to the pipeline as described by Callahan et al. (2016) with minor modifications as outlined by Smith et al. (2020 and 2022). Quality checks of both forward and reverse reads were determined based on the visualization of Q scores with the aim of ensuring mean Q scores of >30 for forward and reverse reads. Filtering and trimming of poor-quality reads and removal of primer sequences were conducted using the trimLeft function in DADA2. Identical sequences were combined using the de-replication function followed by the merging of forward and reverse reads. An Amplicon Sequence Variant (ASV) table was then constructed following which chimeric sequences were removed and taxonomy assigned to sequence variants using the SILVA database (Version 138.1) downloaded from the DADA2 website (https://benjjneb.github.io/dada2/training.html). A bootstrapping threshold of 80 was applied for taxonomic classification by incorporating minBoot = 80 as part of the assigned Taxonomy function as described by Smith et al. (2020). Sample metadata, sequence taxonomy, and ASVs were combined into a phyloseq object using phyloseq (version 1.44.0; McMurdie and Holmes, 2013) for further analysis. To determine the proportion of rumen methanogens belonging to the SGMT or RO clade, ASVs assigned to the *Methanobrevibacter* genus were further classified by conducting an online NCBI BLAST search against the RefSeq database (https://blast.ncbi.nlm.nih.gov/Blast.cgi?PAGE_TYPE=BlastSearch). Only bacterial and archaeal ASVs, classified beyond the phylum level, were obtained. Subsequently, alpha (Shannon) diversity was calculated for each sample. For differential abundance analysis, ASVs which were not present in >20% of the samples and with read counts of <10, were removed before calculating relative abundances.

### Statistical Analysis

Methane emissions, animal performance, diet digestibility, rumen fermentation, and ruminal microbiome were analyzed as a randomized complete block design with a 2 × 2 factorial structure, where the backgrounding growth (optimal vs. suboptimal), finishing diet (forage vs. grain), their interaction, and the pen were considered as fixed effects and block as a random effect. Methane emissions, animal performance, diet digestibility, and rumen fermentation were analyzed using the MIXED procedure of the Statistical Analysis System software (SAS Institute, 2013); meanwhile ruminal microbiome was analyzed using the GLIMMIX procedure of SAS (2013). Normal distribution and equality of variance were checked using the UNIVARIATE procedure and outliers were identified from the studentized residuals. Differences between treatments were determined by F-tests using Type III sums of squares. The PDIFF command incorporating the Tukey test was applied to evaluate pairwise comparisons between treatment means. Results were presented as least square means ± standard error of the mean, differences were considered statistically significant at *P* < 0.05, and tendency at *P* < 0.10.

## Results

### Animal Performance and Methane Emissions

During the backgrounding period, the optimal growth rate steers displayed two-fold higher BW and HCW gains compared to suboptimal growth steers (*P* < 0.01; Table 1). Additionally, DMI was 17% higher (*P* < 0.01) for optimal steers compared to suboptimal steers. Daily CH_4_ emissions tended (*P* < 0.10) to be higher for optimal growth steers in comparison to suboptimal growth steers; however, CH_4_ yield (*P* < 0.10) and Ym factor (*P* < 0.05) were 10% lower for optimal steers compared to suboptimal group. Finally, during the backgrounding period, CH_4_ intensities expressed per unit of BW gain or HCW gain for optimal steers was approximately half (*P* < 0.01) of those obtained for the suboptimal steers. During the finishing period, suboptimal steers had 24% more (*P* < 0.01) BW gain than optimal steers; however, the HCW gain was only 11% higher (Table 1). DMI relative to BW was 8% higher (*P* < 0.01) for previously suboptimal steers. Methane emissions, CH_4_ yield, and Ym factor were not different (*P* > 0.05) as a consequence of the prior backgrounding management. However, CH_4_ intensities expressed per unit of BW gain and HCW gain were 23% and 17% lower (*P* < 0.05) for steers submitted previously to suboptimal backgrounding compared to optimal backgrounded steers. Grain steers had higher (*P* < 0.01) BW gain and HCW gain compared to forage steers (48% and 65%, respectively; Table 1). Methane emissions, CH_4_ yield, and Ym factor were lower (*P* < 0.05) for steers that were managed under grain conditions (27%, 14%, and 17%, respectively). Similarly, methane intensities expressed by BW, BW gain, or HCW gain were also lower (*P* < 0.01) for grain compared to forage-finished steers.

**Table 1.** Performance and methane emissions, by backgrounding growth (optimal vs. suboptimal) and finishing system (grain vs. forage) factors on Angus steers during backgrounding and finishing periods

Item	Backgrounding		Finishing		SEM	*P*-value		
	Optimal	Suboptimal	Grain	Forage		Backgrounding	Finishing	Interaction
Backgrounding period (days 0 to 97)								
Initial BW, kg	446	444	—	—	11	0.475	—	—
Final BW, kg	513	473	—	—	10	<0.001	—	—
BW gain, kg d^−1^	0.69	0.30	—	—	0.03	<0.001	—	—
HCW gain, kg d^−1^	0.41	0.20	—	—	0.01	<0.001	—	—
Intake, kg DM d^−1^	9.7	8.3	—	—	0.2	<0.001	—	—
Intake, % BW	2.02	1.81	—	—	0.03	<0.001	—	—
Daily methane emissions, g CH_4_ d^−1^	215	194	—	—	9	0.066	—	—
Methane yield, g CH_4_ kg DMI^−1^	21.8	23.5	—	—	0.8	0.061	—	—
Ym factor, %	6.79	7.47	—	—	0.23	0.022	—	—
Methane intensity, g CH_4_ kg BW^−1^	0.42	0.41	—	—	0.01	0.472	—	—
Methane intensity, g CH_4_ kg BW gain^−1^	332	639	—	—	45	<0.001	—	—
Methane intensity, g CH_4_ kg HCW gain^−1^	548	941	—	—	41	<0.001	—	—
Finishing period (days 98 to 181)								
Initial BW, kg	513	473	492	495	10	<0.001	0.492	0.629
Final BW, kg	594	574	600	568	14	0.002	<0.001	0.813
BW gain, kg d^−1^	0.97	1.20	1.29	0.87	0.06	<0.001	<0.001	0.459
HCW gain, kg d^−1^	0.73	0.81	0.96	0.58	0.04	0.009	<0.001	0.333
Intake, kg DM d^−1^	11.5	11.8	11.6	11.8	0.3	0.333	0.569	0.128
Intake, % BW	2.09	2.26	2.12	2.20	0.05	0.001	0.097	0.163
Daily methane emissions, g CH_4_ d^−1^	283	261	230	313	13	0.240	<0.001	0.967
Methane yield, g CH_4_ kg DMI^−1^	24.7	23.3	22.2	25.8	1.6	0.467	0.045	0.761
Ym factor, %	7.77	7.34	6.84	8.27	0.50	0.459	0.017	0.777
Methane intensity, g CH_4_ kg BW^−1^	0.49	0.48	0.40	0.56	0.02	0.684	<0.001	0.835
Methane intensity, g CH_4_ kg BW gain^−1^	321	246	188	378	17	0.004	<0.001	0.452
Methane intensity, g CH_4_ kg HCW gain^−1^	432	359	248	543	19	0.016	<0.001	0.435

BW, body weight; HCW, hot carcass weight; DM, dry matter; DMI, dry matter intake.

### Diet Digestibility and Rumen Fermentation

During the backgrounding period, diet DM digestibility and ammonia nitrogen (N-NH_3_) were higher (*P* < 0.01) for steers with optimal growth rates (Table 2). However, there were no differences (*P* > 0.05) in ruminal pH, total VFA, or VFA proportions between the background diets. Additionally, during the finishing period, there were no differences (*P* > 0.05) in diet digestibility, nor any parameters of rumen fermentation measured between previous backgrounding growth treatments. On the other hand, grain-fed steers achieved higher (*P* < 0.01) diet DM digestibility, lower (*P* < 0.01) ammonia nitrogen, and variation (*P* < 0.01) in VFA proportions compared with forage-fed steers. Indeed, an increase (*P* < 0.01) in propionic, lactic, and valeric acids was observed in grain-finished steers, whereas an increase (*P* < 0.01) in acetic and butyric acids, as well as Ac/Pr ratio and (Ac + Bu)/Pr ratio was detected for the forage-finished steers (Table 2).

**Table 2.** Diet DM digestibility, ammonia nitrogen, ruminal pH, total and proportion of volatile fatty acids, by backgrounding growth (optimal vs. suboptimal) and finishing system (grain vs. forage) factors on Angus steers during backgrounding and finishing periods

Item^1^	Backgrounding		Finishing		SEM	*P*-value		
	Optimal	Suboptimal	Grain	Forage		Backgrounding	Finishing	Interaction
Backgrounding period (days 0 to 97)								
Diet DM Digestibility, %	60.6	50.4	—	—	0.6	<0.001	—	—
N-NH_3_, mg/dL	8.4	7.0	—	—	0.4	0.004	—	—
Ruminal pH, 1-14	7.20	7.17	—	—	0.03	0.254	—	—
Lactic acid, mM	0.0	0.0	—	—	0.0	0.073	—	—
Total VFA, mM	72.2	81.9	—	—	3.9	0.067	—	—
Acetic, %	72.2	70.8	—	—	1.5	0.517	—	—
Propionic, %	15.5	14.3	—	—	1.2	0.486	—	—
Butyric, %	11.8	14.5	—	—	1.5	0.179	—	—
Valeric, %	0.5	0.3	—	—	0.1	0.131	—	—
Ratio Ac/Pr	6.2	6.3	—	—	0.3	0.842	—	—
Ratio (Ac + Bu)/Pr	7.5	7.8	—	—	0.4	0.638	—	—
Finishing period (days 98 to 181)								
Diet DM Digestibility, %	76.0	75.4	82.1	69.3	0.5	0.380	<0.001	0.069
N-NH_3_, mg/dL	9.7	8.5	6.7	11.5	0.8	0.289	<0.001	0.777
Ruminal pH, 1-14	6.74	6.81	6.72	6.83	0.04	0.292	0.088	0.638
Lactic acid, mM	3.4	3.7	6.2	0.9	0.9	0.821	<0.001	0.584
Total VFA, mM	108.4	107.6	104.2	111.8	4.8	0.903	0.269	0.931
Acetic, %	49.2	49.6	43.7	55.1	1.0	0.755	<0.001	0.684
Propionic, %	23.5	22.9	24.3	22.0	0.6	0.498	0.014	0.947
Butyric, %	13.0	13.7	11.4	15.3	0.8	0.580	<0.001	0.356
Valeric, %	14.3	13.9	20.6	7.5	1.2	0.774	<0.001	0.785
Ratio Ac/Pr	2.2	2.3	1.9	2.6	0.1	0.590	<0.001	0.613
Ratio (Ac + Bu)/Pr	2.8	2.9	2.4	3.3	0.1	0.505	<0.001	0.821

DM, dry matter; N-NH_3_, ammonia nitrogen; Ac/Pr, acetic/propionic; (Ac + Bu)/Pr, (acetic + butiric)/propionic.

### Rumen Microbial Community

After quality filtering, merging, and removal of chimeric sequences, an average of 64,424 ± 7,860 reads per rumen sample were generated. *Prevotellaceae* was the most abundant bacterial family observed, with a mean relative abundance of 33.5% across all samples. The families *Succinivibrionaceae*, *Lachnospiraceae*, and *Christensenellaceae* contributed to 14.3% of the bacterial community composition. *Prevotella* was the primary genus of bacteria observed with a mean relative abundance of 27.6% across all samples, followed by *Succinivibrionaceae* UCG-001 (4.4%), *Christensenellaceae R-7 group* (3.5%), and *Prevotella_7* (3.5%). The genera *Methanobrevibacter* and *Methanosphaera* accounted for 76.0% and 18.8%, respectively, of the archaeal community across all samples. Within the *Methanobrevibacter* genus, the relative abundance of members of the SGMT and RO clade was 78.6% and 21.4%, respectively.

Rumen samples obtained from steers finished under a forage-based regimen had a greater bacterial and archaeal diversity (*P* < 0.01; Tables 3 and 4) compared with steers fed a grain-based finishing diet. Similarly, diet had a significant impact on the rumen microbiome profile, with the relative abundance of some 29 bacteria and four archaeal genera significantly different (*P* < 0.05) between grain and forage-finished animals. The effect of the finishing diet on the relative abundance of individual rumen bacteria and archaea is displayed in Tables 3 and 4, respectively. No lasting effect of feeding management during the backgrounding period was observed on the diversity of the rumen bacteria and archaea communities during the finishing phase (*P* > 0.05). Equally, minimal impact on the relative abundance of individual rumen bacteria and archaea was observed, with only *Fibrobacter* and *NK4A214 group* genera of bacteria impacted by backgrounding feed management (*P* < 0.05).

**Table 3.** Characterization of the rumen bacterial family and genera (%), by backgrounding growth (optimal vs. suboptimal) and finishing system (grain vs. forage) factors on Angus steers during the finishing period

		Backgrounding		Finishing		SEM	*P*-value		
		Optimal	Suboptimal	Grain	Forage		Backgrounding	Finishing	Interaction
Shannon diversity		5.28	5.17	4.24	6.22	0.09	0.377	<0.001	0.406
Family	Genus								
[Eubacterium] coprostanoligenes group	“unclassified”	1.30	1.03	1.87	0.46	0.14	0.122	<0.001	0.194
Acholeplasmataceae	Anaeroplasma	0.96	0.72	0.27	1.40	0.10	0.078	<0.001	0.663
Acidaminococcaceae	Succiniclasticum	2.70	1.83	3.31	1.21	0.35	0.051	<0.001	0.167
Bacteroidales BS11 gut group	“unclassified”	0.80	0.65	0.01	1.43	0.10	0.307	<0.001	0.304
Bacteroidales RF16 group	“unclassified”	1.54	1.65	0.57	2.62	0.23	0.753	<0.001	0.357
Bifidobacteriaceae	Bifidobacterium	1.03	0.76	1.75	0.04	0.26	0.426	<0.001	0.579
Christensenellaceae	Christensenellaceae R-7 group	3.87	3.20	2.00	5.07	0.42	0.258	<0.001	0.814
Erysipelatoclostridiaceae	Erysipelotrichaceae UCG-002	1.13	0.72	1.85	0.01	0.54	0.599	0.021	0.608
	Sharpea	0.91	0.45	1.36	0.00	0.46	0.436	0.023	0.436
F082	“unclassified”	1.95	1.80	2.12	1.63	0.38	0.768	0.329	0.741
Fibrobacteraceae	Fibrobacter	0.89	1.83	0.42	2.30	0.25	0.012	<0.001	0.014
Lachnospiraceae	“unclassified”	1.42	1.53	1.36	1.59	0.23	0.724	0.480	0.464
	[Ruminococcus] gauvreauii group	2.30	1.42	3.40	0.32	0.66	0.334	0.001	0.378
	Acetitomaculum	1.42	1.35	1.49	1.29	0.26	0.854	0.596	0.847
	Agathobacter	0.35	0.82	1.16	0.00	0.20	0.109	<0.001	0.107
	Lachnospiraceae NK3A20 group	3.06	2.46	2.49	3.03	0.28	0.138	0.172	0.697
Muribaculaceae	“unclassified”	0.72	1.19	1.82	0.09	0.25	0.191	<0.001	0.165
Oscillospiraceae	Colidextribacter	0.65	0.42	1.02	0.05	0.26	0.545	0.012	0.548
	NK4A214 group	1.43	1.02	0.69	1.76	0.13	0.029	<0.001	0.683
Prevotellaceae	Prevotella	28.34	26.92	20.90	34.35	1.45	0.487	<0.001	0.706
	Prevotella_7	2.18	4.82	6.98	0.02	1.19	0.125	<0.001	0.122
	Prevotellaceae UCG-001	1.58	1.60	1.34	1.84	0.23	0.937	0.128	0.361
	Prevotellaceae UCG-003	0.78	0.69	0.39	1.08	0.06	0.298	<0.001	0.678
Rikenellaceae	Rikenellaceae RC9 gut group	2.36	2.08	1.52	2.92	0.19	0.290	<0.001	0.780
Ruminococcaceae	CAG-352	0.47	0.60	1.05	0.02	0.25	0.710	0.006	0.616
	Ruminococcus	2.70	2.50	2.87	2.33	0.50	0.780	0.442	0.818
Saccharimonadaceae	Saccharimonas	0.61	0.56	0.14	1.04	0.06	0.624	<0.001	0.725
Selenomonadaceae	Mitsuokella	0.50	0.75	1.25	0.00	0.22	0.426	<0.001	0.419
Spirochaetaceae	Treponema	0.82	0.91	0.32	1.42	0.08	0.445	<0.001	0.007
Succinivibrionaceae	Succinivibrio	0.90	1.26	2.12	0.04	0.70	0.631	0.008	0.660
	Succinivibrionaceae UCG-001	3.68	5.07	8.72	0.03	1.55	0.529	<0.001	0.515
Veillonellaceae	Megasphaera	1.42	1.58	3.00	0.00	0.40	0.780	<0.001	0.771
“unclassified” Absconditabacteriales (SR1)	“unclassified”	0.72	0.74	0.02	1.44	0.09	0.925	<0.001	0.961
“unclassified” Clostridia UCG-014	“unclassified”	1.39	1.53	1.48	1.44	0.14	0.495	0.830	0.244
“unclassified” Gastranaerophilales	“unclassified”	2.69	3.27	1.55	4.41	0.39	0.296	<0.001	0.346
“unclassified” RF39	“unclassified”	0.73	0.91	0.54	1.10	0.08	0.097	<0.001	0.807

**Table 4.** Characterization of the rumen archaea family and genera (%), by backgrounding growth (optimal vs. suboptimal) and finishing system (grain vs. forage) factors on Angus steers during finishing period

			Backgrounding		Finishing		SEM	*P*-value		
			Optimal	Suboptimal	Grain	Forage		Backgrounding	Finishing	Interaction
Shannon diversity			1.99	2.01	1.73	2.27	0.04	0.664	<0.001	0.711
Family	Genus									
Methanobacteriaceae	Methanobrevibacter		76.41	75.61	84.95	67.07	1.08	0.599	<0.001	0.163
	Methanosphaera		19.34	18.34	14.39	23.29	0.96	0.462	<0.001	0.695
	“unclassified”		0.75	0.96	0.42	1.29	0.13	0.221	<0.001	0.973
Methanomethylophilaceae	Methanomethylophilus		0.03	0.10	0.02	0.12	0.04	0.212	0.103	0.062
	“unclassified”		3.44	4.94	0.20	8.18	0.63	0.102	<0.001	0.106
Methanosarcinaceae	Methanimicrococcus		0.01	0.05	0.01	0.05	0.03	0.330	0.330	0.125
Nitrososphaeraceae	Nitrocosmicus		0.01	0.01	0.01	0.01	0.01	0.862	0.752	0.097
	Methanobrevibacter clades									
		SGMT	81.12	76.04	78.84	78.32	2.31	0.112	0.870	0.191
		RO	18.88	23.96	21.16	21.68	2.31	0.112	0.870	0.191

## Discussion

Improving the overall feed efficiency of ruminant livestock production has the potential to benefit farm profitability and reduce GHG emissions (Taylor et al., 2020; Kearney et al., 2022). The exploitation of CG in growing animals has been shown to improve the growth efficiency of beef cattle (Keogh et al., 2015; Keady et al., 2021). However, prior to this study, there was a dearth of information available on the impact of CG on enteric CH_4_ emissions, rumen fermentation, and digestibility.

Growth rates obtained in our trial during the backgrounding and finishing periods were within the range expected for these diets. Commonly background growth rates fluctuated between 0.2 and 0.8 kg/d (Nicol and Kitessa, 1995; Neel et al., 2007; Ashfield et al., 2014; Correa et al., 2021), while finishing on forage between 0.8 and 1.0 kg/d (Nicol and Kitessa, 1995; Neel et al., 2007; Peripolli et al., 2017), and grain finishing growth rates were higher than 1.3 kg/d (Keogh et al., 2015; Peripolli et al., 2017; Correa et al., 2021). Additionally, the increase in BW gain during the 3 mo of realimentation by the steers with suboptimal backgrounding in our study (0.23 kg/d) is within the range of response reported by several authors (0.18 to 0.46; Nicol and Kitessa, 1995; Yambayamba et al., 1996; Neel et al., 2007; Keady et al., 2021). Estimates of enteric CH_4_ emissions from individual animals in the present study were consistent with those of previous studies assessing the methanogenic output of growing beef cattle. Berndt and Tomkins (2013), Bell et al. (2016), Van Lingen et al. (2019) and Smith et al. (2021) found that daily CH_4_ emissions fluctuate around 160 to 230 g of CH_4_/d, with average CH_4_ yield between 19 and 26 g CH_4_/kg DMI (Hristov et al., 2013b; Bell et al., 2016; Van Lingen et al., 2019; Smith et al., 2021) and average CH_4_ intensities between 170 and 210 g CH_4_/kg BW gain (Benaouda et al., 2019; Smith et al., 2021).

Both the quantity and quality of feed consumed by ruminant livestock are the predominant factors influencing ruminal methanogenesis (Clark et al., 2011; Hristov et al., 2013a, 2018). Indeed, during the backgrounding period, a tentative increase in daily CH_4_ emissions in animals offered an optimal plane of nutrition can be explained by the greater intake for optimally fed steers compared to suboptimally fed steers. However, when CH_4_ emissions were expressed relative to DMI/GEI or per unit of BW/HCW gain, suboptimal animals had an increased CH_4_ output. Greater feed intake or greater quality of ingested feed has been positively related to animal performance (Fox et al., 2004; NASEM, 2016); equally high animal performance is negatively related to CH_4_ intensities (Hristov et al., 2013b; Beauchemin et al., 2020). Thus, as was reported by Smith et al. (2021), we found an inverse relationship between BW gains and CH_4_ intensities, showing the strong association that exists between these two variables.

Across different diet types, both CH_4_ yield and Ym factor have been shown to fluctuate between 6 and 35 g CH_4_/kg DMI (Van Lingen et al., 2019) and between 2% and 12% of the daily GEI (Johnson and Johnson, 1995), respectively. Both NDF and starch content, as well as overall DM digestibility, are the predominant factors associated with dietary-related differences in CH_4_ yield and Ym (Sauvant and Giger-Reverdin, 2009; Hristov et al., 2013a; Jaurena et al., 2015; Arndt et al., 2022). Consistent with previous research, the reduction in CH_4_ yield and Ym factor, observed in steers with optimal growth during the backgrounding period, is likely explained by the lower NDF content and higher DM digestibility of the diet offered during this period, since there is a negative correlation between digestibility and CH_4_ yield (Yan et al., 2009) and between NDF content and CH_4_ yield (Archimède et al., 2011).

During the finishing period, suboptimal backgrounded steers had increased daily gains (BW and HCW). Additionally, CH_4_ intensities expressed by weight gain (BW or HCW) were reduced for suboptimal backgrounded steers. These results agree with results previously highlighted by Hristov et al. (2013b) and Beauchemin et al. (2020) whereby cattle with higher growth rates display decreased CH_4_ emissions intensities. However, this reduction in intensities was explained by their higher growth rate rather than a lower CH_4_ emission per se. Certainly, daily CH_4_ emissions, CH_4_ yield, and Ym factor were not different between optimal and suboptimal growth during the finishing phase, indicating that the previous backgrounding phase did not alter CH_4_ emissions. The lack of differences in diet DM digestibility, rumen fermentation, and microbial community potentially explains the absence of differences in CH_4_ emissions between steers previously backgrounded on optimal or suboptimal diets.

Grain-fed steers displayed increased performance and reduced CH_4_ emissions and intensities relative to forage-fed steers (Berndt and Tomkins, 2013; Hristov et al., 2013a). In our trial, grain-finished steers emitted 80 g less CH_4_ per day compared to forage-finished steers. Diets containing high levels of starch will result in less CH_4_ emissions than those composed mainly of structural carbohydrates (Moss et al., 2000; Lovett et al., 2005). Starch fermentation in concentrate results in more propionic acid than cellulose in forage (Beauchemin et al., 2020). Additionally, diets rich in grain produce a lower concentration of acetic acid and a higher concentration of valeric acid and some organic acid intermediates, like lactic acid (Murphy et al., 1982; Bannink et al., 2006). Ruminal propionic and valeric production are considered a competitive H^+^ sink to methanogenesis, with acetic and butyric acids considered a net contributor to ruminal H^+^ (Bannink et al., 2006; Ellis et al., 2014). In our experiment, grain steers had increased propionic, valeric, and lactic acids and reduced acetic and butyric acids and the ratios Ac/Pr and (Ac + Bu)/Pr; where, Ac/Pr ratio as well as (Ac + Bu)/Pr ratio are recognized indicators of an animal’s methanogenic potential (Moss et al., 2000; Williams et al., 2019).

Consistent with the effects of both the backgrounding and finishing diet on rumen fermentation, effects on the rumen microbial profile across treatments, were predominantly observed by finishing diet. Although changes to the structure of the rumen microbial profile have been observed in feed-restricted animals previously (McCabe et al., 2015), the lack of difference in VFA concentration observed during the backgrounding phase in the present study, is in line with no major differences in the microbial profile of the rumen between optimal and suboptimal steers during the backgrounding period. Additionally, McCabe et al. (2015) and Cristobal-Carballo et al. (2021) found that the rumen microbiota (and associated fermentation end-products) is driven by the diet consumed at the time of sampling and that previous dietary interventions do not lead to a detectable long-term microbial imprint. In our study, similar results to those were found for optimal and suboptimal steers after 2 mo of finishing since there were practically no differences in the microbial communities.

Consistent with previous research, the effect of contrasting diets during the finishing phase (Petri et al., 2013; Henderson et al., 2015; McGovern et al., 2020; Waters et al., 2020; Cristobal-Carballo et al., 2021), altered the composition of the rumen microbiota. Variations in the proportions of structural carbohydrates amongst forage and concentrate-based diets, likely explain differences in the bacterial diversity amongst both diets in the finishing period (Counotte et al., 1981; Ransom-Jones et al., 2012; Petri et al., 2013; Chen et al., 2019; Cristobal-Carballo et al., 2021). Indeed, owing to the enhanced complexity of structural plant carbohydrates (cellulose, lignocellulose, pectin, etc), in comparison to starch, increased diversity of the rumen bacterial community associated with a forage, diversifies the availability of carbohydrates within the rumen, thus supporting a higher quantity of rumen bacteria across various ecological niches (Henderson et al., 2015; McGovern et al., 2020; Waters et al., 2020). Similarly, increases in the relative abundance of starch-degrading bacteria (*Succinivibrionaceae* family and *Succiniclasticum*, *Erysipelotrichaceae UCG-002*, *Sharpea*, and *Megasphaera* bacteria genera) and decreases of fiber-degrading bacteria (*Prevotella*, *Fibrobacter*, *Rikenellaceae RC9*, and *Christensenellaceae R-7* bacteria genus) for steers fed grain-based compared with steers fed forage-based, is consistent with the known fermentative capabilities of individual genera of bacteria observed in the current study. In addition, in our study, we were unable to find differences between *Methanobrevibacter* clades by finishing diets, which might be partially expected since there were no differences in ruminal pH (Reeve et al., 1997; Smith et al., 2022). *Methanosphaera* can produce CH_4_ via the reduction of methanol, and in the rumen, methanol is a product of pectin hydrolysis (Carberry et al., 2014). So, the higher proportion of *Methanosphaera* in our forage diet may have been due to alfalfa having a high pectin concentration (8-10%; NASEM, 2016).

Suboptimal steers had reduced DMI and subsequently CH_4_ emissions during the backgrounding period. However, more days of finishing in either forage or grain diet were required to achieve the same BW as their contemporaries. Therefore, while it may be beneficial to reduce (total) CH_4_ during the backgrounding phase, the effects on the lifetime emissions of the animal and subsequent profitability need to be assessed within the context of an entire beef farm system, considering the different ages at slaughter between feeding strategies.

## Conclusion

Our study on CG under pasture-based beef production systems highlights its potential for CH_4_ emissions mitigation strategies within both forage and grain contexts. In both finishing diets, the lower CH_4_ intensity of steers initially subjected to a suboptimal growth trajectory during the backgrounding period is due to their relatively higher performance during finishing, rather than lower CH_4_ emissions or yield per se. In fact, there were no differences in diet digestibility, rumen fermentation, or the rumen microbiome either under the forage or grain finishing regimens regardless of the prior backgrounded nutritional management of the animals. Lifetime emissions of the steers need to be assessed with the different dietary regimens since suboptimal steers reduced CH_4_ emissions during the backgrounding period; but, additional days of finishing were required to achieve the same BW as their contemporaries.

## Acknowledgments

J.C. was awarded a Teagasc Walsh Scholarship to fund his PhD research studies (grant number N-2018060).The authors acknowledge the financial support by Instituto Nacional de Investigación Agropecuaria (INIA), Uruguay (grant number N-19407).
